# Barriers and enablers to the implementation of the 6-PACK falls prevention program: A pre-implementation study in hospitals participating in a cluster randomised controlled trial

**DOI:** 10.1371/journal.pone.0171932

**Published:** 2017-02-16

**Authors:** Darshini R. Ayton, Anna L. Barker, Renata T. Morello, Caroline A. Brand, Jason Talevski, Fiona S. Landgren, Mayer M. Melhem, Evelyn Bian, Sandra G. Brauer, Keith D. Hill, Patricia M. Livingston, Mari Botti

**Affiliations:** 1 Department of Epidemiology and Preventive Medicine, School of Public Health and Preventive Medicine, Monash University, Melbourne, Victoria, Australia; 2 Project Health, Cremorne, Victoria, Australia; 3 Division of Physiotherapy, School of Health and Rehabilitation Sciences, The University of Queensland, Brisbane, Queensland, Australia; 4 School of Physiotherapy and Exercise Science, Curtin University, Western Australia, Australia; 5 Epworth/Deakin Centre for Clinical Nursing Research, Deakin University, Victoria, Australia; 6 School of Nursing and Midwifery, Deakin University, Burwood, Victoria, Australia; University of Ottawa, CANADA

## Abstract

Evidence for effective falls prevention interventions in acute wards is limited. One reason for this may be suboptimal program implementation. This study aimed to identify perceived barriers and enablers of the implementation of the 6-PACK falls prevention program to inform the implementation in a randomised controlled trial. Strategies to optimise successful implementation of 6-PACK were also sought. A mixed-methods approach was applied in 24 acute wards from 6 Australian hospitals. Participants were nurses working on participating wards and senior hospital staff including Nurse Unit Managers; senior physicians; Directors of Nursing; and senior personnel involved in quality and safety or falls prevention. Information on barriers and enablers of 6-PACK implementation was obtained through surveys, focus groups and interviews. Questions reflected the COM-B framework that includes three behaviour change constructs of: *capability*, *opportunity* and *motivation*. Focus group and interview data were analysed thematically, and survey data descriptively. The survey response rate was 60% (420/702), and 12 focus groups (n = 96 nurses) and 24 interviews with senior staff were conducted. Capability barriers included beliefs that falls could not be prevented; and limited knowledge on falls prevention in patients with complex care needs (e.g. cognitive impairment). Capability enablers included education and training, particularly face to face case study based approaches. Lack of resources was identified as an opportunity barrier. Leadership, champions and using data to drive practice change were recognised as opportunity enablers. Motivation barriers included complacency and lack of ownership in falls prevention efforts. Motivation enablers included senior staff articulating clear goals and a commitment to falls prevention; and use of reminders, audits and feedback. The information gained from this study suggests that regular practical face-to-face education and training for nurses; provision of equipment; audit, reminders and feedback; leadership and champions; and the provision of falls data is key to successful falls prevention program implementation in acute hospitals.

## Introduction

Despite advances in clinical practice and research, falls remain the most common adverse event in hospitals. More than 240,000 in-hospital falls occur each year in England and Wales with falls being the most commonly reported safety incident in National Health Service hospitals [1, 2]. Falls prevention programs for hospitalised older people are multifaceted, reflective of the complex causal pathway for falls. With increased complexity comes increased risk of implementation failure. Implementation of falls prevention programs can be influenced by several factors including environmental and contextual issues; staff knowledge, beliefs and attitudes; organisational culture and climate; staff workloads; and access to appropriate equipment and resources [3]. An understanding of these factors can inform the development of an implementation plan that addresses the barriers and enablers to the implementation of the intervention.

There is limited information about the barriers and enablers to the implementation of falls prevention in acute hospitals. Two survey based studies implemented across five acute care hospitals in Singapore showed that nurses perceived the greatest barriers to implementation of fall prevention practices to be: staff and patient education; lack of motivation in staff; availability of support staff; and access to facilities and equipment [4–5]. These barriers were also reported in a recent Cochrane review of 11 RCTs [6]. Other barriers reported in the review included: leadership support at the organisational and unit level; engagement of front-line staff in program design; pilot-testing to identify potential barriers to implementation; provision of data about falls; and changes in nihilistic staff attitudes about falls prevention were associated with successful implementation of inpatient falls prevention programs in hospitals [6].

Tailoring the implementation of falls prevention programs to the local context optimises implementation. The 6-PACK falls prevention program is nurse-led (Box 1) and was developed as part of continuous quality improvement activities at an Australian acute hospital. An evaluation reported that fall-related injuries appeared to reduce following the implementation of the program [7]. This led to a multi-centre randomised controlled trial (RCT) to further establish the efficacy of the 6-PACK program [8, 9]. Whilst 6-PACK intervention components are required to remain fixed in an RCT, the implementation of the program was tailored to the local context of the intervention wards to ensure implementation was optimised.

**Box 1. The 6-PACK program**

The 9 item fall-risk tool [10] is updated for each patient each shift by their treating nurse. Patients identified as high falls risk receive:

1. A ‘falls alert’ sign positioned above their bed, and one or more of the following interventions:
2. Supervision of patients in the bathroom
3. Ensuring patients’ walking aids are within reach
4. A toileting regime
5. A low-low bed
6. A bed/chair alarm

The COM-B model was developed by condensing concepts from 19 frameworks of behaviour change identified in a systematic review by Michie and colleagues [11]. The COM-B model demonstrates human behaviour (B) as the interaction between physical and psychological capabilities (C) that utilise social and environmental opportunities (O) via motivators (M) that are reflective (‘thinking’ with the head) or automatic (‘thinking’ with the heart). It has been widely adopted in implementation and health services research [12, 13].

The aim of this study was to use the COM-B model to identify the perceived barriers to, and enablers of, implementation of the 6-PACK program from the perspectives of nurses and senior staff to inform the implementation plan. Specifically we sought to identify physical and psychological factors (capability); environmental and social contexts (opportunity); and reflective and autonomic processes (motivation) that are perceived to be barriers or enablers of the successful implementation of the 6-PACK program. In addition, we sought to gain insights into what strategies could be applied to optimise successful implementation of the 6-PACK program in the RCT.

## Materials and methods

### Design

A multi-centre mixed methods study. This study was part of the 6-PACK project that incorporated a three-year research plan: 1) Studies of current falls prevention practice and moderators (pre-implementation) [14]; 2) A cluster RCT testing 6-PACK effectiveness (S1 Appendix), including economic [15] and program evaluations (implementation); and 3) An assessment of sustainability of practice change and outcomes (maintenance). The study reported here forms part of the pre-implementation stage.

### Participants and setting

Detailed information about participants, recruitment and data collection are reported elsewhere (S2 Appendix). In brief, this study involved staff from 16 medical and 8 surgical wards participating in the 6-PACK RCT. Nurses were invited to complete the survey and participate in focus groups. Key informant interviews were conducted with senior staff (Nurse Unit Managers (NUMs), senior physicians, Directors of Nursing (DONs) and clinical services, falls prevention leaders and senior personnel involved in quality, safety and risk management).

### Nurse survey

The 42 item survey was developed with items related to beliefs about falls; current falls prevention practice; 6-PACK program components; best practice guidelines and key recommendations; and reporting practices were included. Participants indicated their level of agreement using a five point Likert scale ranging from strongly disagree to strongly agree. Seven items related to the COM-B domains: one to capability, three to opportunity and three to motivation (Table 1).

**Table 1:** Mapping of survey, focus group and interview questions to the COM-B domains [11].

Survey	Focus group	Interview	Questions/Statements
**Capability: The individual’s psychological and physical capacity to engage in the activity concerned.**			
	✓	✓	What strategies would you recommend we use when implementing the 6-PACK program? Why?
	✓	✓	What learning can we take from other program implementation experiences on your ward? What were some of the barriers? What would you do differently next time? What worked well?
✓			You can’t stop older people from falling.
	✓	✓	Do you believe falls can be prevented? What interventions do you feel are most important?
**Opportunity**: **The factors that lie outside the individual that make the behaviour possible or prompt it.**			
	✓	✓	Who are the critical people that need to be involved in falls prevention activities at your hospital?
	✓	✓	What strategies/factors would you consider to be essential to sustaining programs like the 6-PACK? Please explain.
		✓	What falls prevention activities are currently occurring/or planned for the hospital? Do you perceive these activities to be complementary or inhibitory to the 6-PACK implementation on the intervention wards? Please explain.
		✓	Who should we involve in the processes of implementing the 6-PACK this hospital? What do you see their role will be? How do you rate the relative importance of these individuals or group in terms of making the implementation successful?
		✓	Who do you anticipate may be obstructive/resistive to the implementation of 6-PACK? Why? (Knowledge, beliefs and skills? Attitudes and opinions? Conflicting demands?) What strategies do you recommend to better engage these people? (Incentives and motivators?) What strategies do you recommend to inform/approach/involve key staff in the change process?
		✓	What system level barriers do you feel may exist to implementing the 6-PACK program? E.g. Equipment and staffing resources, communication, leadership and teamwork, environmental constraints (e.g. budgets, redevelopments, restructuring)
✓			Leadership and supervision for falls prevention practice.
✓			An active falls prevention leader is essential for falls prevention programs to be successful on my ward.
✓			This feedback [about how I use falls prevention interventions] helps me use falls prevention interventions more effectively.
**Motivation: Reflective and automatic mechanisms that activate or inhibit behaviour. Includes habitual processes, emotional responding, as well as analytical decision-making.**			
	✓	✓	What effect do you feel audit, feedback and reminders will have on the effectiveness of the 6-PACK program implementation? Can you provide examples of when these have been effectively used previously?
✓			There are more important things I should do than falls prevention interventions for my high falls risk patients.
✓			Incident reporting provides us with a way of measuring how we are going with patient falls.
✓			It is not my responsibility to stop patients from falling.

### Focus groups and key informant interviews

Discussion guides for the focus groups and key informant interviews based on the COM-B framework were developed to elicit ward nurse and senior staff views on barriers and enablers to implementing the 6-PACK program (Table 1). Focus groups and key informant interviews at each hospital were scheduled and conducted. Senior staff nominated by the DON at each hospital received a letter of invitation to participate in an interview from the research team. The perspectives of senior staff were sought to understand hospital practices, policies and the organisational context influencing falls prevention interventions.

### Data analysis

Descriptive statistics were calculated for survey responses using Stata MP v13 statistical software. Analysis of interview and focus group data was continuous with deductive coding being applied for the three COM-B domains and emerging themes explored and tested for applicability and consistency. Three researchers independently coded and recoded transcripts using Nvivo (QSR International 2012), continually working back and forth between data sources in a process of open, axial and thematic coding [16, 17]. Discrepancies were resolved by discussion and consultation with the investigator team as required. Quantitative and qualitative data were analysed separately with a process of triangulation applied at the interpretation stage of the analysis whereby findings from each component were considered to determine whether findings were convergent, complementary or contradictory [18].

### Ethics

This study was approved by Monash University Human Research Ethics Committee–CF11/0229–2011000072 and each of the relevant hospital ethics committees. Participants were given verbal information about the study and asked to sign consent forms if they were interested in participating.

## Results

### Study participants

Overall, 702 surveys were distributed with 420 (60%) returned. The majority of respondents were registered nurses (74%); staff working on medical wards (75%); and staff with at least one year of experience at the hospital (74%). Twelve focus groups involving 96 nurses and 24 interviews with senior staff (SS) were conducted. Six DONs, seven NUMs, one Clinical Risk Coordinator, one Quality and Safety Manager, one clinical program nurse manager, and eight nursing educators participated in the interviews.

Each of the COM-B domains and arising sub-themes are described below (Table 2) in the context of barriers and enablers to the implementation of the 6-PACK program. Implementation strategies suggested by the participants have also been described and summarised in Table 3.

**Table 2:** Mapping of barrier and enabler themes to COM-B domains.

COM-B domain		Theme
Capability	Barrier	• Management of complex patients (N) • Belief that falls are inevitable (N) • Ward layout (N)
	Enabler	• Training and education (N and SS) ■ Face-to-face education ■ Case study based teaching
Opportunity	Barrier	• Lack of resources (N)
	Enabler	• Use of falls data (SS) • Feedback on progress (N) • Competition (SS) • Leadership (SS)
Motivation	Barrier	• Lack of ownership (SS) • Complacency (SS, N)
	Enabler	• Goal to reduce falls (SS) • Engaging staff in falls prevention • Emotional impact of patient falls (N) • Improved patient outcomes (SS) •Audit, reminders and feedback (N and SS)

N = nurses, SS = senior staff

**Table 3:** Strategies to optimise successful implementation of the 6-PACK program

COM-B domain	Rationale	Implementation strategy
Capability	• Improve knowledge and skills • Support attitudinal change • Model new behaviours	• Regular practical face-to-face education and training for nurses (ward walk arounds, small interactive group sessions)
Opportunity	• Provide and discuss data • Inform about progress	• Provision of falls data • Leadership and champions (ward champions, Nurse Unit Managers) • Provision of equipment • Newsletters and posters communicating progress, achievements and stories
Motivation	• Reinforce key strategies for falls prevention • Troubleshoot and provide support • Demonstrate commitment to project	• Compliance audits • Reminders and feedback • Reward and recognise change in practice and leadership

### Capabilities

#### Management of complex patients

Implementing falls prevention interventions was viewed as difficult, particularly when treating patients with complex health issues. A nurse’s ability to manage multiple risks including pressure areas, medications, nutrition and falls was described as a “*daunting balancing act”*.

When you’re looking at those more elderly, confused, aggressive patients, it’s weighing up between the falls risk versus the medication management to keep them settled…it is a balancing act. (SS3, Hospital (H) 3)

#### Belief that falls are inevitable

Many nurses reported that they were unable to prevent falls, despite feeling they had knowledge in falls prevention. They identified a number of patient characteristics that they perceived were associated with high falls risk and not amenable to falls prevention interventions.

We’ve got patients on the ward who are in the high visibility area, on low-low beds, have the pressure sensor, [yet] they are still falling… I don’t think falls can be prevented. (Nurse, H3)

We’ve got dementia patients… You can do as much as you can, and [falls are] still just going to happen…I don’t think falls can be prevented. (Nurse, H3)

Only 46% of nurses responding to the survey disagreed with the statement ‘You can’t stop older people from falling’, while 23% were undecided. This suggests discord in beliefs regarding the inevitability of falls. Senior staff were less likely than nurses to accept the inevitability of falls based on patient characteristics, and emphasised the need to minimise the impact of falls.

[Falls] should be preventable. We shouldn’t have them. I think it’s about changing that perception and that belief, and that awareness, [to be] that actually any fall is wrong, it shouldn’t have happened. (SS2, H6)

#### Ward layout

The layout of the ward was often perceived by nurses as a hindrance to surveillance. Single rooms made it difficult for nurses to physically move efficiently from one patient to another.

Sometimes we have four [high risk] patients in three different rooms, it’s a disaster…how do you get to look at everyone at the same time? (Nurse, H5)

Say if you’re stuck in a room or a bathroom with someone and someone else buzzed…you mightn’t see that [patient] for 20 minutes because you’re in doing a massive dressing or [something else]. (Nurse, H1)

#### Training and education

Improving knowledge and skills through training and education sessions were identified as enablers to falls prevention practice. Survey data indicated only 32% of nurses felt they received useful training from falls prevention leaders. Senior staff valued e-learning methods as they believed that information could be conveyed efficiently.

I put a module of falls strategies on e-Learning so nurses can access information on falls. (SS1, H5)

Nurses raised issues such as lack of access to computers and *“no time to get to the computers*” as a barriers to e-learning education. Nurses specified that although e-learning was convenient, practical and hands-on training on the ward with case studies was preferable to increase their capabilities in falls prevention.

We all prefer face-to-face learning rather than e-learning…I think you learn more with real-life situations. (Nurse, H5)

Senior staff valued ongoing feedback and case review as an effective means of enhancing falls prevention knowledge.

I educate the staff every month about falls we’ve had…I explain strategies that could have been improved. I go and speak to staff who have been involved in a fall and find out why a strategy wasn’t put in place, what were the obstructions to that, and the circumstances around it. (SS3, H3)

In addition to discussions on the delivery mode for education, staff raised suggestions for education content. Nurses identified the specific need for education on the treatment of delirium and management of patients with cognitive impairment. Senior staff highlighted the need for training on how to connect fall-risk tool scores to appropriate interventions.

### Opportunity

#### Access to resources

A key barrier identified in the implementation of the 6-PACK program was access to resources. One of the interventions of the 6-PACK program was to put high risk patients on a low-low bed, however there was not a sufficient number of beds available on the wards for nurses to use. This was further complicated by a lack of tracking systems of where the beds are within a hospital.

We have about 12 low-low beds, which is not sufficient. There is no system of tracking where the Low-Low bed is. The poor nurse has to ring Environmental Services or six different wards to see if they have a Low-Low bed. (SS2, H2)

If I’ve identified someone as a high-falls risk, I’ve got to put an intervention in place, [but] we don’t have the resources [equipment] to do that. (Nurse, H3)

#### Use of data to drive practice change

Senior staff highlighted the need to ensure that nursing staff understood the extent of the problem of falls on the wards. This involved presenting data on the trends and benchmarking of ward falls across wards.

Here’s our data, this is what we’re looking like and your patient safety boards…I think that’s really valuable because it puts your performance up there to be seen as a trend; they can be benchmarking against themselves. (SS3, H1)

The majority of nurses surveyed (75%) agreed with the statement *‘incident reporting provides us with a way of measuring how we are going with patient falls’*. Providing this ***feedback on progress*** in falls prevention to nurses was seen as an opportunity to encourage and promote practice change.

Participants were asked if using data to promote competition between wards would encourage falls prevention action. While senior staff believed *“a bit of competition between wards”* was a good idea, nurses were less positive as they felt ward experiences would vary due to different patient characteristics.

Oh, I don’t think it would make any difference. We’ve all got different patients. (Nurse, H1)

#### Leadership

Leadership, including the establishment of champions for falls prevention was identified as a key enabler for practice change. Leaders were identified by staff as playing a critical role in providing guidance and support to those less experienced, and to develop and promote standardised practices in terms of implementing falls prevention interventions. Nurses were either neutral (35%) or agreed (42%) that there was strong leadership support for falls on their ward and that their supervisors have assisted them when issues of falls have been raised (64%). Senior staff reported that the NUM has a critical role in falls prevention.

The NUMs are important players in [falls prevention]…to educate staff and support them about the right techniques. (SS3, H3)

NUMs were also seen as vital in ensuring the sustainability of the program.

[NUMs] are going to be the drivers, not just from the beginning but in six months’ time when it’s implemented. (SS1, H3)

Champions were identified as a practice change strategy for other projects including infection control, pain management and wound care. They were able to provide a link between committees, senior management and the ward staff and provide education and support while on the wards.

The falls champion on that ward will play a very active role in delivering the education and doing the assessments …because that links back to the Falls Committee. (SS3, H4)

Senior staff emphasised that the key to a successful champion is finding staff who have *“the passion for falls and wants to make a difference to patient care”* and willing to push the agenda of falls prevention on the wards. One staff member described champions as ‘*resource people*’.

### Motivation

#### Lack of ownership

A perceived barrier to the implementation of the 6-PACK program was a lack of ownership for falls prevention in some hospitals.

Who drives falls? Nobody owns falls. (SS3, H2)

The majority of nurses (80%) believed that they were responsible for falls prevention. Senior staff agreed that nurses were primarily responsible but recognised the value of multidisciplinary input into falls prevention.

It’s everyone’s responsibility to work together to reduce falls. But I suppose primarily it comes back to nurses as they’re there with the patient 24/7. (SS1, H1)

#### Complacency

Reflecting on previous and current falls prevention practice, staff recognised that one barrier to practice change was complacency. Complacency was often discussed in relation to the completion of fall-risk tools. Prior experience of staff suggested that complacency in completing these tools could be an issue with nurses stating “*we all just go tick*, *tick*, *tick*, *tick”*.

Staff just tick the same boxes that were done yesterday without really assessing…There’s that difficulty of just that complacency of ticking the same boxes…that doesn’t give you the best outcome. (SS1, H3)

To address issues of complacency, ***audits*, *reminders and feedback*** were suggested by staff.

Better to be reminded to do this, and reminded all the time. (Nurse, H6)

The other thing that we have a gap in is that we don’t do regular auditing…It’s about the audits and the feedback that’s given. (SS1, H1)

#### Falls prevention goals and commitment

An enabler to falls prevention was a commitment to falls prevention by senior staff demonstrated through provision of resources (equipment and staff) as well as clearly articulated goals. Participants believed this provided motivation and was also a source of pride and achievement when progress was being made.

So it’s pride in falls, reduction in falls. Commitment by staff. And it’s commitment by management…if they’re going to have the need for low-low beds or whatever you need, [they will get it]. Implementation care is paramount. (SS2, H3)

#### Engaging staff in falls prevention

As highlighted by one senior staff participant, staff engagement is important and can be facilitated through ‘*engaging hearts and minds’—*both the emotional and logical aspects of falls prevention. Nurses described feeling ‘*guilty’*, ‘*stressed’* and ‘*distressed’* when a patient under their care experienced a fall. They also described the ‘*worry’* experienced if a patient suffered a fall-related injury. The ***emotional impact of a patient fall*** was seen as something that could be a motivating factor. A senior staff member at one hospital highlighted that nurses responded to interventions that ***emphasised the benefit to the patient***. This also had implications for sustaining the project long term.

If you always promote it as best for the patient and patient focused you’ll get staff on-board, and continuing to help drive the program. You’ve got to be able to sell it to them…first of all say this is going to be so much better for your patient outcomes. (SS1, H1)

## Discussion

This study identified a number of implementation targets, particularly in the areas of motivation and opportunity. These included education and training to address skills, knowledge and beliefs of nurses and developing systems to encourage falls prevention practice such as audits, reminders and feedback, provision of equipment and facilitating a culture of falls prevention through leadership and champions. Previous studies have also reported the above enablers [4–6]. Unlike prior research, this study details differences between nurses and senior staff beliefs regarding falls prevention. Learnings from this study were used to develop an implementation plan for the RCT [8].

The belief in the inevitability of falls is consistent with findings from other studies [6, 19]. Although survey results suggest nurses thought falls could be prevented, nurses in focus groups identified patient groups where they believed falls could not be prevented. There was disagreement between nurse and senior staff perspectives as to whether in-hospital falls could be prevented. Incongruity between nurses’ and senior staff perceptions of the inevitability of falls has implications for the success of a falls prevention program. If nurses do not believe falls can be prevented, it may be difficult to implement interventions that aim to prevent falls. Senior staff recommended that education and training was required to increase nurse confidence and knowledge in how to prevent falls and to utilise the resources provided effectively.

Education was identified as a strategy to improve capabilities. However, implementation did raise some practical challenges. While both senior staff and nurses valued face to face case studies, senior staff favoured e-learning due to convenience and efficiency. Carefully designed e-learning packages can be effective in disseminating best practice education and have the potential to reach less accessible night and casual staff [20]. However, if a model of e-learning was adopted it would be important to ensure nurses have adequate access to computers and that these packages address aspects of falls prevention that are of greatest concern to nurses.

A motivator identified by senior staff was to harness the emotional impact of falls, for example through ‘story telling’ of falls incidents at handover. Case studies with patient stories and experiences of falls may also prove powerful in highlighting the need to address in-hospital falls in education sessions. Communicating clearly the patient benefits of the 6-PACK program was also seen to be a strategy to enhance engagement by staff.

A challenge to motivation is complacency in falls prevention practice. The acute setting is a crowded landscape of patient safety initiatives that can compete for the attention and time of nurses. Previous research has described the phenomenon of ‘missed care’ or ‘unfinished care’ where nurses can find it difficult to achieve all their tasks in caring for a patient. This can lead to adverse patient events such as falls [21]. To promote continuing engagement in strategies and to assist in care prioritisation, senior staff and nurses highlighted the importance of regular audits, reminders and feedback. Audits, reminders and feedback are generally an effective approach in guiding the implementation of an intervention [22]. Providing data to demonstrate the extent of the problem of falls on wards and to benchmark progress was another strategy identified by participants. Incident reporting has also been identified as a useful approach to change the attitudes, perceptions and practice of staff and promote engagement in patient safety initiatives [23].

The majority of falls prevention programs are focused on nurses and nursing interventions with falls often considered a nursing sensitive patient outcome [24]. However, a barrier to motivation identified is the lack of ownership for falls prevention. Senior staff stated that falls prevention should involve a multidisciplinary team approach and is everyone’s responsibility. Conversely, a recent study in Australian hospitals reported that doctors perceived time limitations as a major barrier to their involvement in falls prevention and acknowledged that medical priorities were more important for them [25]. While 6-PACK is a nurse led program, it does not discourage involvement of other clinicians. Indeed, nurses are a critical link between the patient and other care team members and often are responsible for communicating on progress and changes in patient status. This importance of the nurse role in multi-disciplinary management of falls should be communicated to staff in training.

The opportunity domain examined factors outside of the individual which enable or prompt falls prevention action. The key themes related to opportunity included lack of availability of resources, provision of falls data and leadership for falls prevention. The lack of availability of falls prevention equipment, such as low-low beds, has previously been described [19, 24]. Leadership is both an opportunity and motivation strategy and was recognised as important by both nurses and senior staff. NUMs and champions were identified as key individuals in the implementation and sustainability of falls prevention interventions. The need for leadership and champions has been reported as an important implementation strategy in the literature [6, 26, 27].

### Limitations and future research

The 6-PACK program is a nurse delivered intervention and therefore the focus of this research was to seek the perspective of nursing staff. The perspectives of other health professionals (doctors, allied health professionals such as physiotherapists, occupational therapists) involved in direct patient care were not captured in this study. Further research to explore whether the barriers and enablers identified by nurses and senior staff are also identified by other hospital staff is required. The wards that participated in this study volunteered to take part in the 6-PACK RCT which may have introduced selection bias. This may have impacted on the results with participants being more likely to recognise the importance of falls prevention practice.

## Conclusions

This study identified barriers and enablers to the implementation of the 6-PACK program corresponding to the constructs of capability, opportunity and motivation. Barriers identified included beliefs that falls could not be prevented; limited knowledge on falls prevention in patients with complex care needs (e.g. cognitive impairment); lack of resources; and lack of ownership in falls prevention efforts. Enablers included education and training, particularly face to face case study based approaches; improved leadership; using data to drive practice change; and use of reminders, audits and feedback. Successful falls prevention program implementation in acute hospital wards are likely to require a multifaceted, planned approach that includes: regular practical face-to-face education and training for nurses to modify skills and established beliefs; provision of equipment; audit, reminders and feedback; leadership and champions; and the provision of falls data.

## Supporting information

S1 Appendix 6-PACK programme to decrease fall injuries in acute hospitals: cluster randomised controlled trial (published article). (PDF)

S2 Appendix Development of an implementation plan for the 6-PACK falls prevention programme as part of a randomised controlled trial: protocol for a series of preimplementation studies (published article). (PDF)
